# Regulation of rat HspB5/alphaB-Crystallin by microRNAs miR-101a-3p, miR-140-5p, miR-330-5p, and miR-376b-3p

**DOI:** 10.1007/s12192-023-01371-8

**Published:** 2023-08-16

**Authors:** Britta Bartelt-Kirbach, Nikola Golenhofen

**Affiliations:** Institute of Anatomy and Cell Biology, University of Ulm, Albert-Einstein-Allee 11, 89081 Ulm, Germany

**Keywords:** HspB5/αB-crystallin, Small heat shock protein, MicroRNA, Cellular stress, Neuroprotection

## Abstract

HspB5/alphaB-crystallin is an ubiquitously expressed member of the small heat shock protein family which help cells to survive cellular stress conditions and are also implicated in neurodegenerative diseases. MicroRNAs are small non-coding RNAs fine-tuning protein expression mainly by inhibiting the translation of target genes. Our earlier finding of an increase in HspB5/alphaB-crystallin protein amount after heat shock in rat hippocampal neurons without a concomitant increase of mRNA prompted us to look for microRNAs as a posttranscriptional regulatory mechanism. Microarray miRNA expression data of rat hippocampal neurons under control and stress conditions in combination with literature search, miRNA binding site prediction and conservation of target sites yielded nine candidate microRNAs. Of these candidates, five (miR-101a-3p, miR-129-2-3p, miR-330-5p, miR-376b-3p, and miR-491-5p) were able to convey a downregulation by binding to the HspB5 3′- or 5′-UTR in a luciferase reporter gene assay while one (miR-140-5p) led to an upregulation. Overexpression of these six microRNAs in C6 glioma cells showed that three of them (miR-101a-3p, miR-140-5p, and miR-376b-3p) regulated endogenous HspB5 protein amount significantly in the same direction as in the reporter gene assay. In addition, overexpression of miR-330-5p and miR-491-5p in C6 cells resulted in regulation of HspB5 in the opposite direction as expected from the luciferase assay. Analysis of miRNA expression in rat hippocampal neurons after cellular stress by qPCR showed that miR-491-5p was not expressed in these cells. In total, we therefore identified four microRNAs, namely miR-101a-3p, miR-140-5p, miR-330-5p, and miR-376b-3p, which can regulate rat HspB5 directly or indirectly.

**Supplementary Information:**

The online version contains supplementary material available at 10.1007/s12192-023-01371-8.

## Introduction

HspB5/alphaB-crystallin belongs to the family of small heat shock proteins (HspBs) which are vitally important for cells to survive stress conditions as heat, hypoxia, or oxidative stress as they bind partially unfolded proteins and keep them in a refolding-competent state (Bakthisaran et al. 2015). In addition, HspB5 has anti-apoptotic, anti-inflammatory and neuroprotective functions (Acunzo et al. 2012; Rothbard et al. 2012; Zhu and Reiser 2018). The relevance of its neuroprotective function is visible in many neurological and neurodegenerative diseases. For example, HspB5-deficient mice show an increased lesion size after experimental stroke (middle cerebral artery occlusion, MCAO), an effect which could be reversed by intraperitoneal injection of recombinant HspB5 (Arac et al. 2011). Furthermore, HspB5 is also found upregulated and associated with pathological protein aggregations of several neurodegenerative diseases such as Morbus Alzheimer or Parkinson (Jellinger 2000; Wilhelmus et al. 2006). HspB5 overexpression is beneficial in a mouse model for Huntington disease (Oliveira et al. 2016). All these data show the need for a better understanding of the molecular mechanisms of regulation and function of HspB5 in the brain. It might be a promising target for developing new therapeutic strategies for neurological diseases which are a major cause of death and disability worldwide.

During our earlier studies on the upregulation of HspBs after stress in rat hippocampal neurons we discovered that HspB5 protein amount was regulated post-transcriptionally, as it increased significantly after heat shock while the mRNA amount remained constant (Kirbach and Golenhofen 2011). This prompted us to look for microRNAs as possible regulators of HspB5. MicroRNAs are non-coding RNAs of approximately 22 nt (nucleotides) length which bind mostly to the 3′-UTR (untranslated region) of protein coding mRNAs and inhibit their translation. Important for miRNA binding is a “seed” sequence of 6-8 nt at the 5′ end which binds with 100% complementarity to the target sequence. The remaining miRNA sequence has no exact match with the target sequence which makes it difficult to reliably predict mRNA targets from the miRNA sequence alone. This feature of miRNA binding also accounts for one microRNA having multiple mRNA targets and thus serving for the fine-tuning of the expression of concordant genes. MicroRNAs are also involved in brain development and function and are dysregulated in various diseases of the central nervous system (CNS) (Hussein and Magdy 2021; Tsujimura et al. 2022; Li et al. 2023).

We started this study with a microarray expression analysis to identify potential miRNA candidates for the regulation of HspB5 and combined the results with in silico target prediction as well as determination of binding site homology between rat, mouse, and human. The resulting nine miRNA candidates were then further analyzed for their potential to directly regulate HspB5 after stress.

## Materials and methods

### Cell culture

Primary rat hippocampal neurons were isolated as described previously from Sprague-Dawley rat embryos at gestational stage E19 (Bartelt-Kirbach et al. 2016). Timed pregnant Sprague-Dawley rats (RjHan:*SD*, RRID: RGD_38676310) were obtained from Janvier Labs (Le Genest-Saint-Isle, France) and housed in the local animal facility under a 12 h day/night cycle with food and water ad libitum until experiments. Sacrifice of embryos and organ removal for neuron culture was approved by the local ethics committee (University of Ulm) under the ID number O.103-8. All animal experiments were performed in compliance with the guidelines for the welfare of experimental animals issued by the Federal Government of Germany, the National Institute of Health and the Max Planck Society. Neurons were seeded at a density of 30,000 cells/cm^2^ in 6 cm petri dishes and cultivated in Neurobasal medium (ThermoFisher Scientific) supplemented with B27 and 1 μM glutamine for 14 days without medium change in an incubator at 37 °C, 5% CO_2_, 95% humidity.

C6 (rat glioma, RRID:CVCL_0194) and HEK293 (human embryonic kidney, RRID:CVCL_0045) cell lines were cultivated in DMEM with 10% FBS (ThermoFisher Scientific).

### miRNA profiling

At day in vitro (DIV) 14, primary rat hippocampal neurons were subjected either to heat shock (42 °C, 30 min) or sodium arsenite stress (100 μM, 30 min). Total RNA was isolated 0 h (heat shock) and 6 h (sodium arsenite) after stress and from untreated controls with the NucleoSpin miRNA kit (M+N) according to the manufacturer’s instructions. RNA was sent to Febit biomed GmbH (Heidelberg, Germany) for miRNA profiling on a Geniom® Biochip MPEA rattus norvegicus, covering miRBase v. 14.0 (http://www.mirbase.org/; Griffiths-Jones et al. 2008) The array contains 15 intra-array replicates/miRNA (Baum et al. 2003; Vorwerk and Roberts 2008). The raw intensity data was background corrected and normalized using VSN (variance stabilizing normalization, Huber et al. 2002), the resulting median values were used to calculate the median fold change as a ratio of two groups (qmedian).

### In silico search for putative miRNA binding sites in HspB5-mRNA

An *in silico* search for miRNA binding sites in the HspB5 5′- + 3′-UTR of rat, mouse and human was conducted with mirWalk (Dweep et al. 2011) in combination with seven other algorithms (DIANAmT, miRanda, miRDB, PICTAR4 + 5, PITA, RNA22, RNAhybrid, and Targetscan). miRNAs identified by at least two algorithms and downregulated after stress were then investigated for homology of the target sites between rat, mouse, and human. Only those miRNAs with conserved target site were considered as candidates. Alignment of candidate miRNAs to the HspB5-mRNA sequence was carried out with DiAlign (https://www.genomatix.de/cgi-bin/dialign/dialign.pl; Genomatix Software GmbH).

BLAST (Basic Local Alignment Search Tool; https://blast.ncbi.nlm.nih.gov/Blast.cgi) was used to elucidate the rat miR-491-5p sequence. To this end, a nucleotide BLAST search with the human miR-491-5p sequence was conducted on the rat genomic sequence.

### Luciferase reporter gene Assay

#### Cloning of reporter gene vectors

Two luciferase reporter gene vectors were constructed, one carrying the HspB5-5′-UTR upstream of the luciferase gene, the other harboring the HspB5-3′-UTR downstream of the luciferase gene. HspB5-5′-UTR was amplified from rat muscle cDNA with the primers HspB5-5′-UTR-fw: 5′ GGG GGA TCC AGA CCC TGT CCT GGC TCC AGA GAA CAA G and HspB5-5UTR-rev: 5′ TTG GGG ATC CGA TGG CTA GAT GAG TGT AGA GTC GGT TAG with 35 cycles at 50.3 °C. The PCR product of 348 bp was cleaved with BamHI (restriction sites underlined) and ligated upstream of the luciferase gene into the pMIR-Report™ vector (Ambion, ThermoFisher Scientific). HspB5-3′-UTR was amplified from rat heart cDNA with the primers HspB5-3UTR-fw: 5′ TTC CGA GCT CAT TCC CTT TCC TCG TTG CAT and HspB5-3UTR-rev: 5′ GGG GAC GCG TGT TGC TGA ACG ATA TTT TTT ATT AGC with 5 cycles of 40 °C annealing followed by 30 cycles with 58 °C annealing temperature. The resulting PCR product of 157 bp was cleaved with MluI and SacI (resctriction sites underlined) and ligated downstream of the luciferase gene into the pMIR-Report™ vector. Success of cloning was controlled by sequencing.

#### Transfection of HEK293 cells

HEK293 cells were seeded at 20,000 cells/cm^2^ in 24-well-plates and transfected with 1.5 μl Lipofectamine 3000 (ThermoFisher Scientific), 400 ng pMIR-Report vector, 100 ng pMIR-betaGal control vector, and 10 pmol of miRVana miRNA mimic (Ambion, ThermoFisher Scientific #4464066) or mimic negative control (Ambion, ThermoFisher Scientific #4464058) per well. The following miRNA mimics were tested: hsa-miR-21-5p (ID MC10206), hsa-miR-96-5p (ID MC10422), hsa-miR-129-2-3p (ID MC10076), hsa-miR-330-5p (ID MC11180), mmu-miR-376b-5p (ID MC12738), rno-miR-376b-3p (ID MC12314), and hsa-miR-491-5p (ID MC11479) for HspB5-3′-UTR and hsa-miR-101a-3p (ID MC11414) and hsa-miR-140-5p (ID MC10205) for HspB5-5′-UTR. Cells were lysed 48 h after transfection with reporter lysis buffer (Promega) for the subsequent activity assays.

#### Luciferase assay

To measure the luciferase activity, the Luciferase Assay System (Promega) was used according to the manufacturer’s instructions. Light emission was measured with a Fluoroskan Ascent FL (ThermoFisher Scientific) or a Cytation 3 (BioTek Instruments) multiplate reader.

#### beta-Galactosidase assay

Activity of the beta-Galactosidase encoded by the control vector was measured to correct for transfection efficiency. For this, the β-Galactosidase Enzyme Assay System with Reporter Lysis Buffer (Promega) was used according to the manufacturer’s instructions.

Luciferase activity values were then divided by the respective beta-Galactosidase activity values and subsequently normalized to the value obtained after transfection with the miRNA control mimic.

### RNA isolation, cDNA synthesis, and real-time PCR

For measurement of candidate miRNA amount after stress, rat hippocampal neurons at DIV14 were subjected either to heat shock (42 °C, 30 min) or sodium arsenite stress (50 μM, 30 min). Cells were lysed at different timepoints after stress (0 h, 2 h, 4 h, 7 h, 24 h) with 600 μl lysis buffer and RNA isolated with the miRVana miRNA isolation kit (Ambion, ThermoFisher Scientific) according to the manufacturer’s instructions. C6 cells were subjected to sodium arsenite stress (100 μM, 30 min) and lysed 24 h after stress. Unstressed controls were lysed in parallel. Ten nanogram RNA was then reverse transcribed using the TaqMan™ Advanced cDNA Synthesis Kit (ThermoFisher Scientific). Amplification was carried out with TaqMan™ Fast Advanced Mastermix and TaqMan™ Advanced microRNA Assays (ThermoFisher Scientific). miR-331-3p and miR-484 were selected as TaqMan™ Advanced miRNA control assays. They were previously determined to be the two most stably expressed under stress by the GeNorm algorithm (Vandesompele et al. 2002) and the geometric mean of their expression was used as reference. A custom TaqMan assay was used for the putative rno-miR-491-5p. Real-time PCR was carried out in a LightCycler 1.0 (Roche) with 95 °C, 20 s followed by 40 cycles of 95 °C, 1 s and 60 °C, 20 s. In parallel, 150 ng total RNA was reverse transcribed using SuperScript III (ThermoFisher Scientific) as described before (Kirbach and Golenhofen 2011). Expression of HspB1 together with reference genes CycA and Rpl13A was then measured with QuantiTect SYBR Green PCR kit (Qiagen) and the following PCR protocol: 95 °C, 15 min followed by 45 cycles of 94 °C, 15 s; 55 °C, 30 s; and 72 °C, 30 s to ascertain proper induction of the stress response (see Kirbach and Golenhofen 2011, for details).

### Transfection of miRVana miRNA mimics

C6 cells were seeded in 6-well-plates at a density of 300,000 cells/well 1 day prior to transfection. Transfection of 10 μM miRVana mimics (Ambion, ThermoFisher Scientific #4464066; mimic negative control: #4464058) was carried out with Lipofectamine RNAiMax (ThermoFisher Scientific). The following mimics were used: hsa-miR-129-2-3p, rno-miR-376b-3p, hsa-miR-101a-3p, hsa-miR-140-5p, hsa-miR-491-5p, and hsa-miR-330-5p. In detail, 3 μl mimic was mixed with 150 μl serum-free OptiMEM and 9 μl RNAiMax reagent was diluted in another 150 μl OptiMEM. Both solutions were mixed, incubated for 5 min at room temperature and then 250 μl mixture/well added to the cells. Twenty-four hours after transfection cells were subjected to sodium arsenite stress (100 μM sodium arsenite, 30 min) to induce heat shock protein expression. Cells were lysed with 1.5 × Laemmli buffer with DTE (dithioerythritol) 24 h after stress.

### Western blot

Cell lysates were sonicated and incubated for 5 min at 95 °C. Protein amount of samples was assessed by amidoblack staining (Heinzel et al. 1965). Samples were separated on a 15% SDS polyacrylamide gel and subsequently transferred to a Hybond nitrocellulose membrane (GE Healthcare) using a Mini TransBlot™ chamber (BioRad). Membranes were blocked with 1% low fat milk in TBS (pH 7.4)/0.05% Tween-20 for anti-HspB5 primary antibody (Abcam ab76467, diluted 1:500) and with 2.5% milk for anti-GAPDH primary antibody (ThermoFisher Scientific, MA5-15738, diluted 1:10 000). Primary antibodies were incubated with the membranes overnight at 4 °C. Incubation with the respective secondary antibodies (horseradish-peroxidase conjugated goat-anti-rabbit or goat-anti-mouse IgG, Jackson ImmunoResearch, 111-035-144 and 115-035-068, diluted 1:10 000) was carried out at room temperature for 30–60 min. For signal detection the enhanced chemiluminescence system (Pierce™ ECL Plus substrate, ThermoFisher Scientific) was used. Band intensity was quantified with ImageJ software (National Institute of Health, USA).

### Statistical analysis

The nonparametric Mann-Whitney *U*-test was used for statistical analysis. Statistical significance was assumed for *p* < 0.05.

## Results

### Candidate microRNAs for HspB5 regulation

To select possible candidate microRNAs which might regulate HspB5 protein translation in neurons miRNA expression in cultured rat hippocampal neurons, miRNA binding site prediction and conservation of target sites was investigated.

#### Microarray expression profiling

A microarray expression analysis was conducted with RNA from rat hippocampal neurons under control conditions and under conditions known to lead to upregulation of HspB5, i.e., heat shock and sodium arsenite stress, on a microarray incorporating probes for 388 mature rat miRNA sequences (Online resource 1). The expression change after stress compared to control was evaluated and revealed 22 microRNAs downregulated more than twofold after heat shock and 58 microRNAs downregulated more than twofold after sodium arsenite stress. Seven microRNAs were downregulated more than twofold after both stress conditions, three additionally more than fivefold after at least one stress condition (Online resource 2). Of these ten microRNAs only five (miR-140-5p, miR-376b-5p, miR-455-5p, miR-743a-3p, and miR-802-5p) had putative binding sites in the HspB5 3′- or 5′-UTR and were therefore candidates for its regulation.

#### MicroRNA target prediction

In parallel, we conducted an in silico search for microRNAs with potential target sites in the HspB5-3′ or 5′-UTR using MirWalk, which combines serveral target prediction algorithms. This yielded seven microRNAs predicted by more than 3 algorithms and 45 microRNAs whose target sites were predicted by at least two algorithms (Online resource 3). Of the latter, we excluded all microRNAs showing no downregulation (< 0.7) after at least one stress condition. This resulted in a total of 28 candidate microRNAs in addition to the ones identified by array data. One candidate, miR-743a-3p, was identified by both approaches.

#### Candidates from literature

Two microRNAs, miR-101a-3p and miR-491-5p, were additionally included in the pool of candidates because of hints from the literature. miR-101a has been shown to regulate Alzheimer-related APP as well as a Hsp90 co-chaperone (Vilardo et al. 2010; Liu et al. 2012). Human miR-491-5p has recently been shown to target HspB5 in osteosarcoma (Wang et al. 2017). As this microRNA has no rat equivalent in miRBase, we conducted a BLAST homology search with the human miR-491-5p sequence to the rat genome. From this, a putative rno-miR-491-5p was deduced.

#### Homology of binding sites

In total, our combined approaches thus yielded 34 candidate microRNAs. To further reduce this number, we checked for the quality and conservation of the putative binding sites. Evolutionary conservation is another hint that a microRNA has a functional relevance. The predicted microRNA binding sites were aligned to the HspB5 mRNA sequence of rat, mouse, and human. As the seed region is crucial for miRNA function, it was assessed if this region was conserved between the three species. If the homologous seed region was below 6 nt, the microRNA was removed from the list of candidates.

By this approach, we finally selected nine candidate microRNAs for further functional analysis (Table 1).

**Table 1:**
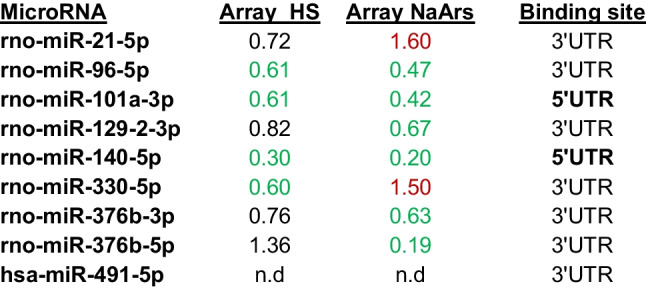
Candidate microRNAs for HspB5 regulation with putative binding sites in the rat HspB5 3′- or 5′-UTR

Array data for heat shock (HS) and sodium arsenite (NaArs) shows qmedian values (upregulation > 1.4 in red or downregulation < 0.7 in green)

*n.d.* not detected, *rno* rattus norvegicus

**Fig. 1:**
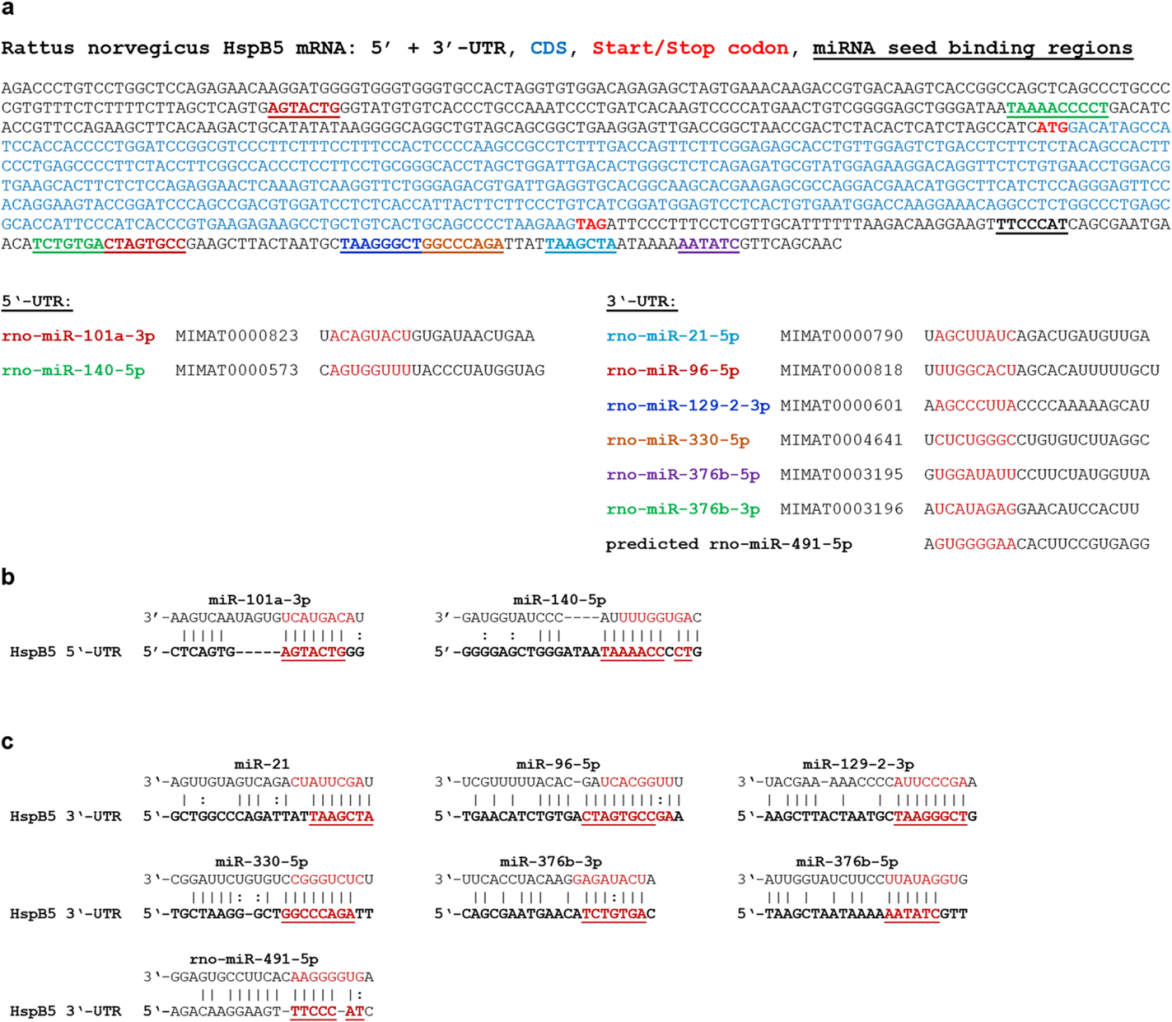
**a** Rat HspB5 mRNA sequence with candidate miRNA binding sites. miRNA seed binding regions on the mRNA are color-coded and underlined. Candidate microRNAs are shown with their sequences and miRBase accession numbers. The seed region of the miRNA sequences is highlighted in red. Two candidate miRNAs can bind to the HspB5 5′-UTR, seven to the 3′-UTR. **b** Alignment of the candidate miRNA sequences to the HspB5 5’-UTR. **c** Alignment of the candidate miRNA sequences to the HspB5 3′-UTR. miRNA seed regions are highlighted in red and additionally underlined in the HspB5 sequence

**Fig. 2:**
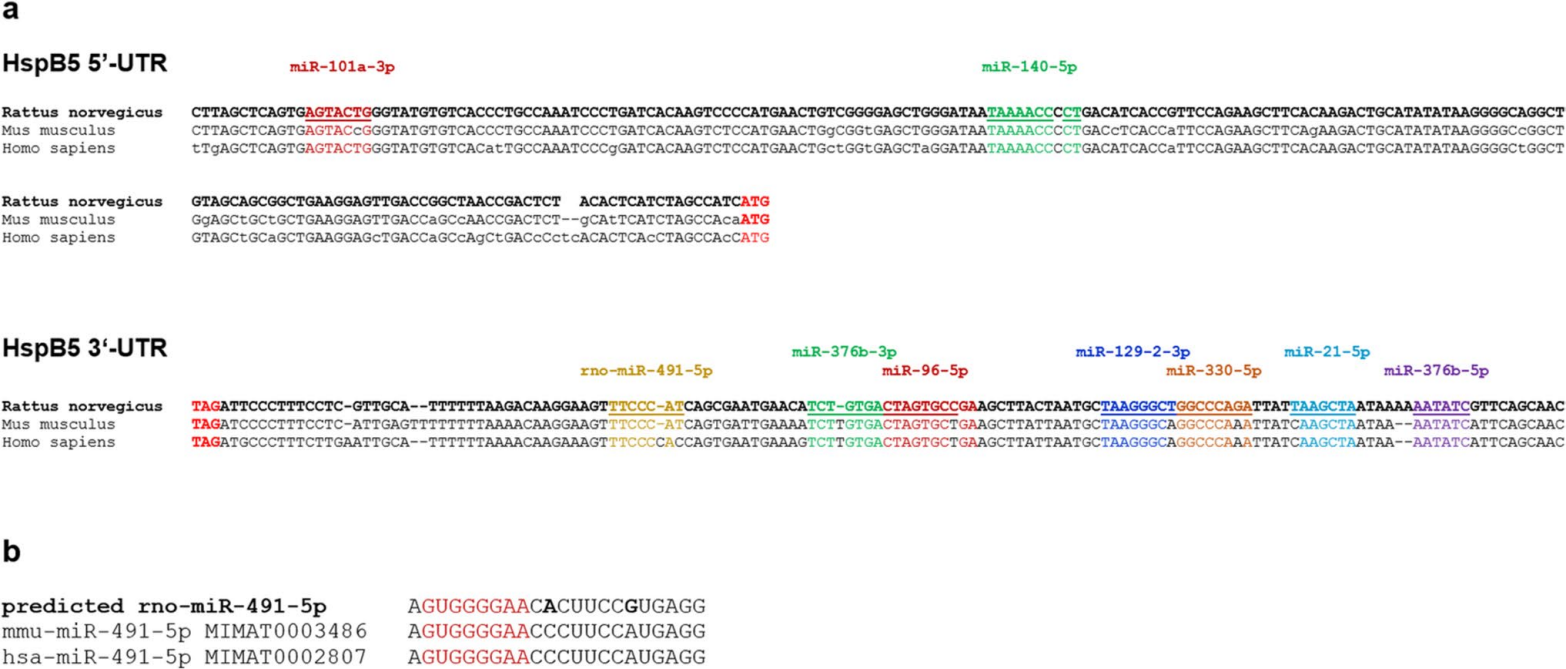
**a** Homology of candidate miRNA binding sites. Homology between rat, mouse and human HspB5-UTR sequence is shown for all candidates. **b** Putative rno-miR-491-5p sequence and homology to human and mouse miR-491-5p. miRNA seed region is highlighted in red. rno, rattus norvegicus; mmu, mus musculus; hsa, homo sapiens

The candidate microRNA sequences and their respective putative binding sites in relation to the HspB5 mRNA are depicted in Fig. 1. Two candidate miRNAs (miR-101a-3p and miR-140-5p) have binding sites in the 5′-UTR, the other seven in the 3′-UTR of HspB5. In addition, the alignment of the candidates with either the HspB5 5′- or 3′-UTR is shown in this figure. Figure 2 depicts the homology of the binding sites between rat, mouse, and human.

The seed binding site for human miR-491-5p, which had recently been described to regulate HspB5 in osteosarcoma cells, shows a 1 nt deletion in the mouse and rat sequence but still has a length of 7 nt and is therefore most likely functional (Fig. 2b).

### Activity of candidate miRNAs in luciferase reporter gene assay

**Fig. 3:**
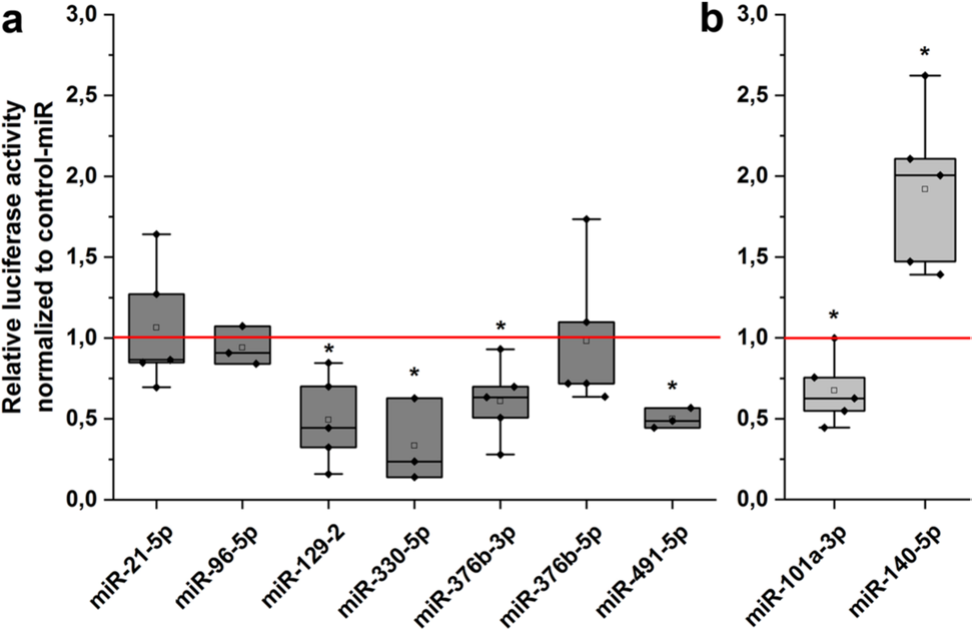
Influence of candidate microRNAs on the HspB5 UTR. Luciferase reporter gene assay with **a** HspB5-3′-UTR and **b** HspB5-5′-UTR. Luciferase activity was normalized to control-miR (set to 1, red line). *n* = 3-5, significance vs. control-miR was assumed for *p* < 0.05, Mann-Whitney *U*-Test. Boxes represent the range from the 25th to the 75th percentile, the thick line within the box represents the median, the open square the mean. Whiskers represent the maximum/minimum value

To assess if the nine candidate microRNAs are indeed able to bind to the HspB5 UTR, we conducted a luciferase reporter gene assay. Transfection of HEK293 cells with miRNA mimics together with a luciferase reporter gene vector carrying either the HspB5-3′-UTR (Fig. 3a) or the HspB5-5′-UTR (Fig. 3b) showed that four candidate miRNAs (miR-129-2-3p, miR-330-5p, miR-376b-3p, and hsa-miR-491-5p) negatively regulated the luciferase activity via the HspB5-3′-UTR while one candidate miRNA, miR-101a-3p, negatively regulated it via the HspB5-5′-UTR. Interestingly, one of the candidates, miR-140-5p, was able to upregulate the luciferase activity via binding to the HspB5-5′-UTR.

### Effect of overexpression of candidate miRNAs on endogenous HspB5

**Fig. 4:**
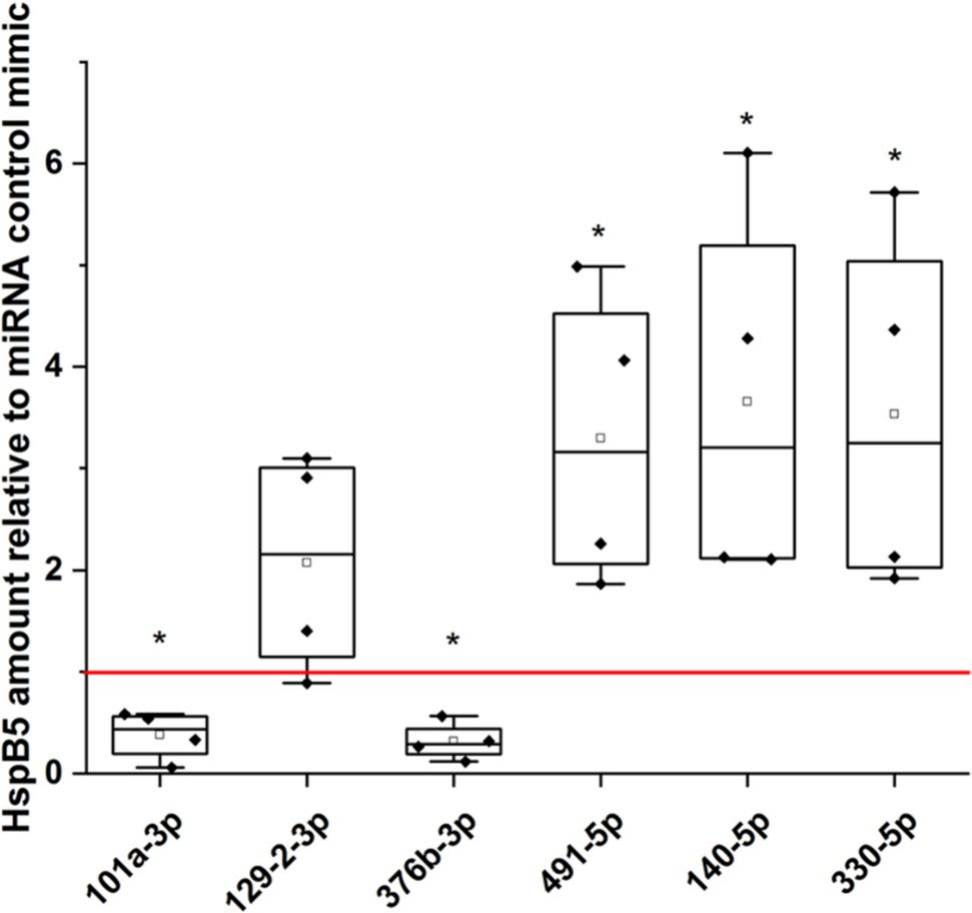
Influence of candidate microRNAs on endogenous HspB5 protein amount. Densitometric measurement of western blots against HspB5 protein in C6 rat glioma cells transfected with miRNA mimics after sodium arsenite stress. HspB5 protein amount was first normalized to GAPDH amount and then to the value of the control mimic (set to 1, red line). *n* = 4, significance assumed for *p* < 0.05, Mann-Whitney *U*-Test. Boxes represent the range from the 25th to the 75th percentile, the thick line within the box represents the median, the open square the mean. Whiskers represent the maximum/minimum value

To investigate if these six candidate microRNAs identified by the luciferase reporter gene assay to bind to the HspB5 UTR are able to regulate endogenous HspB5, we transiently transfected the respective oligonucleotide mimics into rat C6 glioma cells and measured their influence on the amount of HspB5 protein. Since binding of the respective miRNAs to the HspB5 mRNA is mostly expected to lead to a protein downregulation and the constitutive HspB5 amount is undetectable by western blot in C6 cells we upregulated HspB5 amount via sodium arsenite stress to be able to detect a possible downregulation by transfection of miRNA mimics. This stress condition was previously shown to reliably upregulate HspB5 protein amount in these cells. To ensure that the expression of the endogenous miRNAs was not significantly altered in C6 cells by the sodium arsenite treatment alone we measured the respective miRNAs levels 24 h after sodium arsenite stress by qPCR (online resource 4). miRNA expression levels of miR-101a-3p, miR-129-2-3p, miR-140-5p, and miR-330-5p were not significantly altered while miR-376b-3p and miR-491-5p were not expressed in C6 cells Thus, their endogenous microRNA levels cannot be relevant for regulating HspB5 protein amount in the transient transfection experiments. The miRNA levels after transfection were not measured by qPCR, as this was shown to greatly overestimate the amount of functional mimics in the cells because the majority is retained in vesicles and therefore not accessible for the miRNA induced silencing complex (miRISC)(Thomson et al. 2013). Thus, only the functional outcome of the miRNA transfection on HspB5 level was assessed. Western blots for HspB5 of stressed and transfected cells were subjected to densitometric analysis. HspB5 protein levels were first normalized to GAPDH amount measured in parallel to guarantee equal protein loading and second to HspB5 amount of cells transfected with the mimic negative control. Transfection with miR-101a-3p and miR-376b-3p mimics clearly led to a reduced HspB5 amount while transfection with miR-140-5p, miR-491-5p and miR-330-5p mimics significantly increased HspB5 amount (Fig. 4).

Taken together, the effect of overexpression of miR-101a-3p, miR-140-5p, and miR-376b-3p fit to the data from the luciferase assay and, thus, these three microRNAs were identified to directly target HspB5 mRNA and thereby regulate HspB5 protein level. In contrast, overexpression of miR-129-2-3p, miR-330-5p and miR-491-5p showed either no regulation of endogenous HspB5 or regulation in the exact opposite direction as was expected from the luciferase assay data. Therefore, these effects cannot be explained by binding of these miRNAs to HspB5 mRNA, rather another additional mechanism must be responsible for their effect on HspB5 protein level. Most likely these miRNAs have additional targets by which their effect on HspB5 mRNA was neutralized or even reversed. Thus, miR-330-5p and miR-491-5p upregulate HspB5 by an indirect mechanism rather than downregulate it via direct binding to HspB5 mRNA. Table 2 summarizes the effects of the respective microRNAs on HspB5 levels and indicates the direct or indirect regulation mode.

**Table 2:**
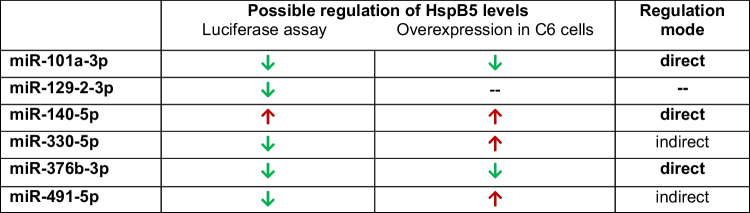
Summary of the data on the regulation of HspB5 by microRNAs

Action of the microRNAs regulating HspB5 in the different assays and their expression after stress. Downregulation is depicted by a green downwards arrow, upregulation by a red upwards arrow

### Expression of miRNA candidates after stress in rat hippocampal neurons

**Fig. 5:**
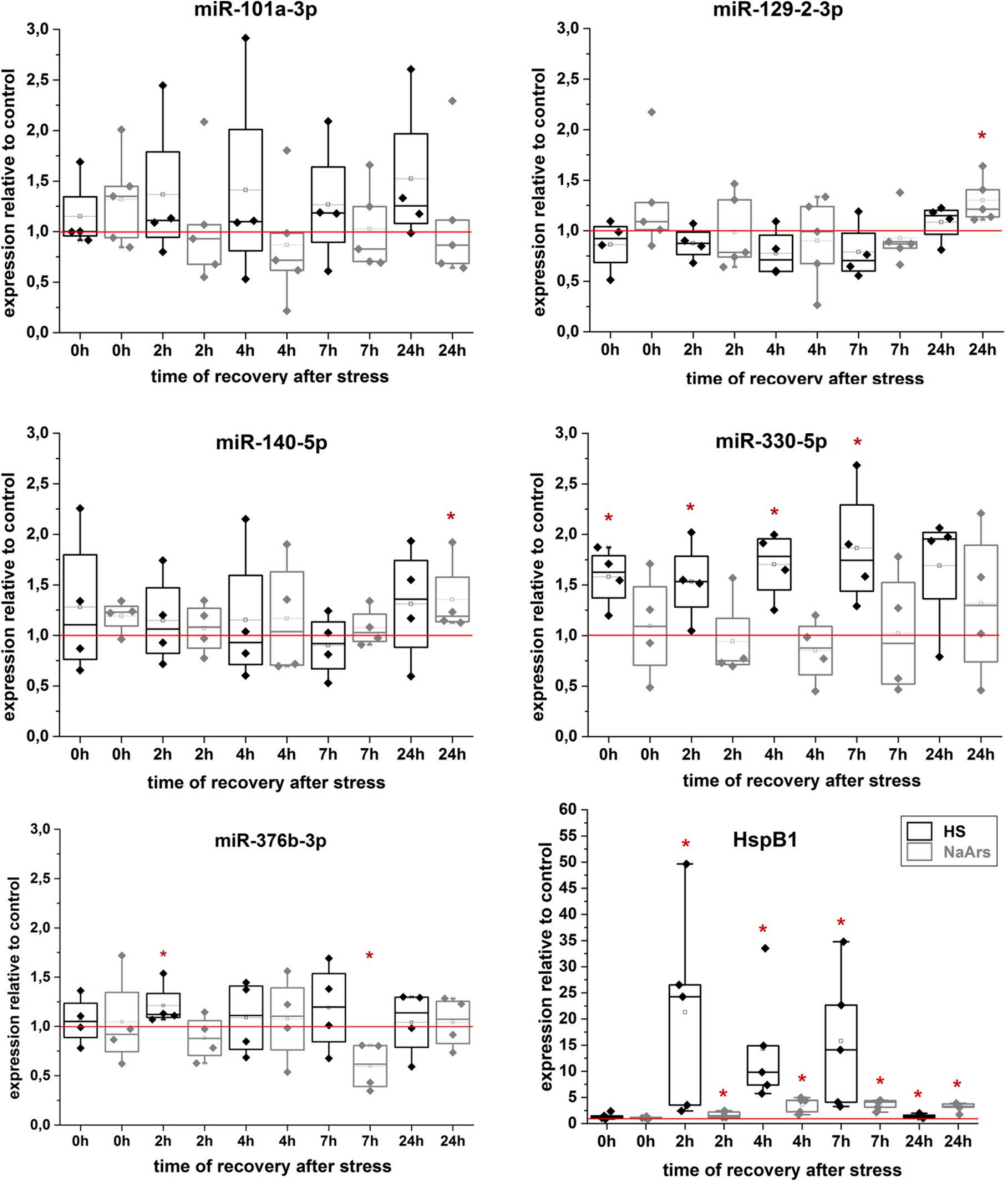
miRNA and HspB1 mRNA expression in rat hippocampal neurons after stress. Expression was measured by real-time RT-PCR after heat shock (black boxes, *n* = 4) or sodium arsenite stress (grey boxes, *n* = 4) relative to unstressed controls (set to 1, red line). Significance was assumed for *p* < 0.05, Mann-Whitney *U*-Test. Boxes represent the range from the 25th to the 75th percentile, the thick line within the box represents the median, the open square with the thin line the mean. Whiskers represent the maximum/minimum value

Since we are interested in microRNAs which may contribute to the upregulation of HspB5 after stress in neurons, we next investigated the expression profile of these six miRNA candidates in detail after two kinds of sublethal stress (heat shock and sodium arsenite stress) in cultured rat hippocampal neurons. As the initial microarray was conducted only with one sample at one timepoint for each stress condition (0 h for heat shock and 6 h for sodium arsenite stress) we now evaluated different timepoints of recovery after stress by real-time RT-PCR in multiple samples (Fig. 5). The selected stress conditions reportedly lead to upregulation of HspB5 protein (Kirbach and Golenhofen 2011; Bartelt-Kirbach and Golenhofen 2014). The mRNA amount of HspB1 was measured in parallel as an internal control to ensure an adequate level of stress because HspB5 mRNA amount does not increase after heat shock. The putative rno-miR-491-5p was not expressed at all in these cells. Thus, this miRNA can be ruled out for upregulation of HspB5 after stress in rat hippocampal neurons.

We found no significant regulation of miR-101a-3p after either stress condition. miR-129-2-3p and miR-140-5p were significantly upregulated 24 h after sodium arsenite stress but not after heat shock. miR-330-5p, however, was significantly increased after heat shock from 0 to 7 h but did not change after sodium arsenite stress. miR-376b-3p was significantly upregulated 2 h after heat shock but significantly downregulated 7 h after sodium arsenite stress (Fig. 5). Interestingly, this detailed analysis of the time course of expression of the candidate microRNAs does mostly not fit to the results of the single microarray experiment initially performed to screen for suitable miRNA candidates (compare with Table 1). This might be due to the fact that the microarray as a screening approach was performed just once and that high variations between individual experiments could be observed as judged from the real-time RT-PCR experiments. Thus, primary neuronal cultures might be very vulnerable and react differently to stress-dependent microRNA expression depending on the batch of culture. In the end, only miR-330-5p showed a significant and relevant upregulation after heat shock (median between 1.5- and 2-fold) whereas expression of the other miRNAs despite some significant differences at certain time points might be of minor relevance.

Taking together the results of the expression profiles, the luciferase reporter gene assay and the microRNA overexpression experiments we found one microRNA, miR-330-5p, which was consistently upregulated after heat stress in cultured neurons and which was able to upregulate HspB5 protein by an indirect regulation mechanism as shown by overexpression experiments in C6 cells. Thus, miR-330-5p might be involved in the reported upregulation of HspB5 in hippocampal neurons after heat shock. In addition, we identified three further microRNAs whose expression was not relevantly affected in hippocampal neurons by two cellular stress protocols but which are able to directly interact with HspB5 mRNA and thereby regulate HspB5 protein level as shown by the overexpression experiments in C6 cells. miR-101a-3p and miR-376b-3p downregulated HspB5 protein amount while miR-140-5p upregulated HspB5 protein amount.

All of these microRNAs might be important under different pathological conditions or in neurological diseases in humans since they are able to interact with HspB5 mRNA and influence HspB5 protein level.

## Discussion

MicroRNAs are important regulators for the fine-tuning of gene expression. One microRNA regulates several genes, mostly with similar or synergistic functions. They are involved in various physiological processes playing key roles in development and cellular differentiation. Furthermore, miRNAs seem to be important in the cellular stress response. Cellular stress-induced downregulation as well as upregulation of certain miRNAs has been described (Mendell and Olson 2012). Stress can alter miRNA biogenesis, mRNA target expression and RISC activity (Leung and Sharp 2010) and the respective miRNAs thereby may counteract adverse conditions and initiate a protective gene expression pattern. Various stress factors, especially oxidative stress, also play a role in the development of neurodegenerative diseases (Sazonova et al. 2021) and, not surprisingly, miRNA dysregulation has also been associated with such diseases (Li et al. 2023). Small heat shock proteins (HspBs) are chaperones crucial for cell survival under such pathophysiological stress conditions. Upregulation of HspBs has been found to be beneficial in neurological and neurodegenerative diseases (Ousman et al. 2007; Sharp et al. 2008; Crippa et al. 2010; Arac et al. 2011; Lee et al. 2012; Tóth et al. 2013), making them interesting therapeutic targets. Interestingly, two HspBs (HspB1 and HspB6) have already been identified as targets of miRNAs (Ren et al. 2009; Choghaei et al. 2016). In this study, we could identify several microRNAs that were able to influence the protein amount of the neuroprotective small heat shock protein HspB5 and, thus, may play a role in neurological diseases or display neuroprotective acitivity.

### Regulation mechanism of miRNAs influencing HspB5 protein level

We found six microRNAs which were able to bind to the HspB5 3′- or 5′-UTR and thereby regulate translation of the luciferase reporter gene. However, miRNA overexpression in C6 cells revealed that only three of them regulated endogenous HspB5 in the same direction as in the luciferase assay. miR-101a-3p and miR-376b-3p led to a HspB5 downregulation in both assays while miR-140-5p conveyed an upregulation in both assays. These three microRNAs are therefore direct regulators of HspB5 protein amount.

MicroRNAs mostly downregulate their target gene expression. To achieve this, the mature miRNA forms a complex with Argonaute proteins called miRNA-induced silencing complex (miRISC). This complex usually binds to the 3′-UTR of the mRNA and initiates either mRNA degradation or translational repression (Orang et al. 2014). However, meanwhile it is recognized that microRNAs, especially those binding to the 5′-UTR of their target mRNAs, can also convey an upregulation of translation through various mechanisms, either by translational activation or by relief of repression (Vasudevan 2012; Orang et al. 2014; Sadakierska-Chudy 2020).

In addition to the three microRNAs directly regulating HspB5 we identified two more, miR-330-5p and miR-491-5p, which are able to bind to the HspB5 mRNA but regulate endogenous HspB5 in C6 cells in the opposite direction as expected. This can only be explained by an indirect regulation mode, meaning that they also bind to other target mRNAs whose regulation then leads to a stimulatory effect on HspB5 expression. This indirect upregulation has to be stronger than the downregulating effect of direct binding of the respective miRNA to the HspB5 mRNA. The sixth microRNA, miR-129-2-3p, did not change endogenous HspB5 protein amount despite being able to bind to the HspB5 mRNA.

### Possible neuroprotective function of the miRNAs directly targeting HspB5

The three microRNAs, miR-101a-3p, miR-140-5p and miR-376b-3p, identified in this study directly targeting and regulating HspB5 are strong candidates to exert neuroprotective activity. Until now little is known about their function in the human organism. Our data showed that miR-101a-3p downregulated HspB5, thus, downregulation of this microRNA would lead to upregulation of HspB5 and probably additional other genes and may thereby result in neuroprotection. Indeed, miR-101a-3p has been shown to be downregulated in Alzheimer patients and in an Alzheimer mouse model (Hébert et al. 2008; Li et al. 2019). It has been shown to directly regulate the amyloid-β precursor protein (APP) and Ran-binding protein 9 (RanBP9) as well as MAPK1, which are all implicated in the pathology of Alzheimer’s disease (Long and Lahiri 2011; Barbato et al. 2014; Li et al. 2019). The expression of miR-101a-3p is also reduced under hypoxic conditions in several cell types (Sun et al. 2015; Zhao et al. 2015) and after 2 h oxygen-glucose deprivation in rat cortical neurons as well as in CNS tissue after experimental ischemia/reperfusion injury (Guo et al. 2021; Zhang et al. 2022) pointing to an impact in stroke. In our study, we saw a downregulation of miR 101-3p in hippocampal neurons after heat shock and sodium arsenite stress in the microarray experiment, however, could not verify this result in the qPCR analysis.

Just as miR-101a-3p, miR-376b-3p directly targeted HspB5 mRNA and downregulated protein level. miR-376b-3p is specifically enriched in neurons (Jovičić et al. 2013) and highly expressed during early neuronal differentiation (Pons-Espinal et al. 2017). Little is known about its regulation during neuropathological conditions, merely a downregulation of miR-376b-3p 14 days after spinal root avulsion in spinal cord has been reported (Tang et al. 2014). In the current study, we observed an upregulation shortly after heat shock but a downregulation 7 h after sodium arsenite stress in the qPCR experiments. Therefore, it is conceivable that its role differs with stress condition, time course and cell type. Whether this microRNA is involved in neuroprotection and may act via targeting HspB5 in human diseases needs further investigation.

The third microRNA, miR-140-5p, identified to regulate HspB5 directly acts in an unusual way, namely upregulating HspB5 protein level by interacting with HspB5 mRNA (see above). Thus, to exert a possible neuroprotective activity by upregulation of HspB5 one would expect an upregulation of this microRNA during neuropathological conditions. In fact, miR-140-5p is upregulated in a mouse model of stroke (Liang et al. 2019) and elevated in the blood of patients with stroke (Sørensen et al. 2014) or late-onset post stroke depression (Liang et al. 2019). In addition, it was upregulated in the hippocampus of Alzheimer disease patients where it acts on ADAM10 (Akhter et al. 2018) and it seems to be a biomarker for cellular senescence (Gullett et al. 2020) as well as for several neurodegenerative diseases (Nguyen et al. 2022). Furthermore, experimental overexpression of miR-140-5p attenuated neuroinflammation and apoptosis in the brain after intracerebral hemorrhage or ischemia via TLR4 of the NFκB pathway (Wang et al. 2019; Song et al. 2021). Thus, miR-140-5p seems to act neuroprotective after ischemic injury in the brain. Since HspB5 is known to play an important protective role in ischemic/reperfusion injury, it is likely that miR-140-5p function is at least partially mediated by HspB5 regulation.

### Possible neuroprotective function of the miRNAs indirectly regulating HspB5

The two microRNAs miR-330-5p and miR-491-5p, which indirectly regulate HspB5 protein amount, are no less interesting than the other three regarding a possible neuroprotective role. Especially our data showing a significant upregulation of miRNA-330-5p after heat shock in cultured hippocampal neurons indicates that this microRNA might contribute to heat shock-induced upregulation of HspB5. miRNA-330-5p has also been reported to be upregulated in certain neuropathological conditions. It is upregulated in dorsal root ganglia of diabetic rats (Guo et al. 2018), after ischemia/reperfusion injury in mice (Zuo et al. 2021) and in the cerebral cortex of Huntington patients (Johnson et al. 2008). Furthermore, it was shown to influence spine number in murine cortex and hippocampal neurons (Cai et al. 2017) and predicted to regulate a network of genes involved in the plasticity and development of hippocampal neurons (Cohen et al. 2014). This would be in line with our hypothesis that miR-330-5p might act neuroprotective by indirectly targeting HspB5. It is unclear via which target mRNAs this indirect regulation could be transmitted as the known validated targets for miR-330-5p have no direct link to HspB5 regulation. HspB5 is upregulated by several transcription factors, the most important being heat shock factor 1 (HSF1)(De Thonel et al. 2012). Among validated target genes for miR-330-5p that might be relevant in the CNS are the NMDA receptor GRIN2A (Yan et al. 2022) and the glial glutamate transporter GLT-1 (Li et al. 2022). GRIN2A is known to activate the PI3K/Akt pathway under stress conditions (Wu and Tymianski 2018). This pathway has been shown to positively regulate heat shock transcription factor 1 (HSF1) (Chatterjee et al. 2013), which could then upregulate HspB5. The glial glutamate transporter GLT-1 is important for removal of extracellular glutamate, a common neurotransmitter known to be neurotoxic in higher concentrations (Rimmele et al. 2021). If this transporter is downregulated this would lead to higher extracellular glutamate levels and might thereby activate stress related pathways in the CNS.

miR-491-5p was found to be not expressed in rat hippocampal neurons as well as in C6 rat glioma cells. However, human miR-491-5p was described previously to regulate HspB5 in human osteosarcoma (Wang et al. 2017). In brain, downregulation of miR-491-5p after trauma ameliorates apoptosis and oxidative stress via upregulation of MT2 (Tang et al. 2022) and a higher serum level of miR-491-5p is associated with a poorer outcome in ischemic stroke patients (Song et al. 2022). These effects of miR-491-5p cannot be explained by acting via HspB5 since our data point to an upregulation of HspB5 by miR-491-5p and HspB5 is known to be protective in ischemic/reperfusion injury.

### Possible synergistic effect of microRNAs

It is well known that one microRNA can regulate multiple target mRNAs, but on the other hand also several different microRNAs can regulate one target mRNA. However, it has been shown that multiple target sites for different microRNAs within the 3′-UTR of one mRNA do not lead to enhanced repression. A synergistic effect multiplying repression has only been described if the target sites for different microRNAs are located with the 3′ and 5′-UTR (Gam et al. 2018). Transferred to our case, this could apply to miR-101a-3p binding to the 5′-UTR and miR-376b-3p binding to the 3′-UTR of HspB5, which both act to repress HspB5 translation.

An additive effect of one microRNA directly regulating a gene and other microRNAs indirectly regulating the same gene (e.g., via transcription factors) has also been reported (Kulyté et al. 2014). This could be the case for miR-140-5p, directly upregulating HspB5, and miR-330-5p upregulating HspB5 protein amount via regulation of a yet unknown pathway. Such additive or synergistic effects would be of interest to explore in the future and remain to be elucidated.

### miRNAs as therapeutic targets

In conclusion, we identified four rat microRNAs, miR-101a-3p, miR-140-5p, miR-330-5p, and miR-376b-3p, which were able to regulate the amount of HspB5 either by direct binding to HspB5 mRNA or indirectly via a yet unknown pathway. These four microRNAs are known to be differentially expressed under various neuropathological conditions. Thus, one might hypothesize that they regulate specific gene patterns including HspB5 and are involved in neuroprotection. Increased HspB5 has been shown to be beneficial in several pathological conditions including neurodegenerative diseases (Rothbard et al. 2012; Zhu and Reiser 2018). Upregulation of HspB5 could therefore be an interesting new therapeutic approach to treat such diseases. Whereas therapeutic upregulation of the HspB5 gene expression in the brain is very challenging, it would be far easier to artificially regulate microRNA expression leading to increased HspB5 levels. Several miRNA therapeutics using viral or non-viral delivery strategies are already being tested in clinical trials for various diseases (Holjencin and Jakymiw 2022; Kim and Croce 2023). Thus, activating specific neuroprotective gene patterns by microRNAs may be an interesting novel therapeutic approach to treat neurodegenerative diseases, even though this might still be a dream of the future as the use of microRNAs as therapeutics also poses many challenges (Diener et al. 2022).

### Supplementary information

ESM 1 (XLSX 62 kb)

ESM 2 (PDF 51 kb)

ESM 3 (XLSX 21 kb)

ESM 4 (PDF 128 kb)
