# Supplementary material for: Regulation of rat HspB5/alphaB-Crystallin by microRNAs miR-101a-3p, miR-140-5p, miR-330-5p, and miR-376b-3p
Source: Cell Stress Chaperones. 2023 Aug 16;28(6):787–99. doi: 10.1007/s12192-023-01371-8 (PMC10746672; doi:10.1007/s12192-023-01371-8)
Supplement: Supplementary file 2 — (PDF 51 kb) [file 12192_2023_1371_MOESM2_ESM.pdf]

Regulation of rat HspB5/alphaB-Crystallin by microRNAs miR-101a-3p, miR-140-5p, miR-330-5p and miR-376b-3p

Cell Stress and Chaperones

Britta Bartelt-Kirbach and Nikola Golenhofen, Institute of Anatomy and Cell Biology, University of Ulm, Albert-Einstein-Allee 11, 89081 Ulm, Germany. britta.bartelt@uni-ulm.de

Online Resource 2: miRNAs downregulated after stress in microarray expression analysis. miRNAs downregulated more than twofold (<0.5) after heat shock (HS) and sodium arsenite (NaArs) stress or more than fivefold (<0.2) after one stress in rat hippocampal neurons and location of their predicted binding site in the HspB5-mRNA (CDS - coding sequence; UTR - untranslated region).

	**change relative to control**		**location of**
	**HS**	**NaArs**	**binding site**
**rno-miR-22-5p**	0.55	0.12	CDS
**rno-miR-140-5p**	0.30	0.20	**5'UTR**
**rno-miR-144-3p**	0.39	0.39	none
**rno-miR-146a-5p**	0.37	0.34	CDS
**rno-miR-181a-1-3p**	0.63	0.17	none
**rno-miR-376a-5p**	0.48	0.41	CDS
**rno-miR-376b-5p**	1.36	0.19	**3‘UTR**
**rno-miR-455-5p**	0.48	0.15	**3'UTR**
**rno-miR-743a-3p**	0.39	0.47	**5'UTR**
**rno-miR-802-5p**	0.48	0.44	**3'UTR**
