# Supplementary material for: Regulation of rat HspB5/alphaB-Crystallin by microRNAs miR-101a-3p, miR-140-5p, miR-330-5p, and miR-376b-3p
Source: Cell Stress Chaperones. 2023 Aug 16;28(6):787–99. doi: 10.1007/s12192-023-01371-8 (PMC10746672; doi:10.1007/s12192-023-01371-8)

Regulation of rat HspB5/alphaB-Crystallin by microRNAs miR-101a-3p, miR-140-5p, miR-330-5p and miR-376b-3p

Cell Stress and Chaperones

Britta Bartelt-Kirbach and Nikola Golenhofen, Institute of Anatomy and Cell Biology, University of Ulm, Albert-Einstein-Allee 11, 89081 Ulm, Germany. britta.bartelt@uni-ulm.de

Online Resource 4: miRNA expression in C6 rat glioma cells 24h after sodium arsenite stress. Expression of the candidate microRNAs was measured by real-time RT-PCR after sodium arsenite stress (n=3) relative to unstressed controls (set to 1, red line). Significance was assumed for $p<0.05$, Mann-Whitney-U-Test. Boxes represent the range from the 25th to the 75th percentile, the thick line within the box represents the median, the open square the mean. miR-376b-3p and miR-491-5p were not expressed in these cells.

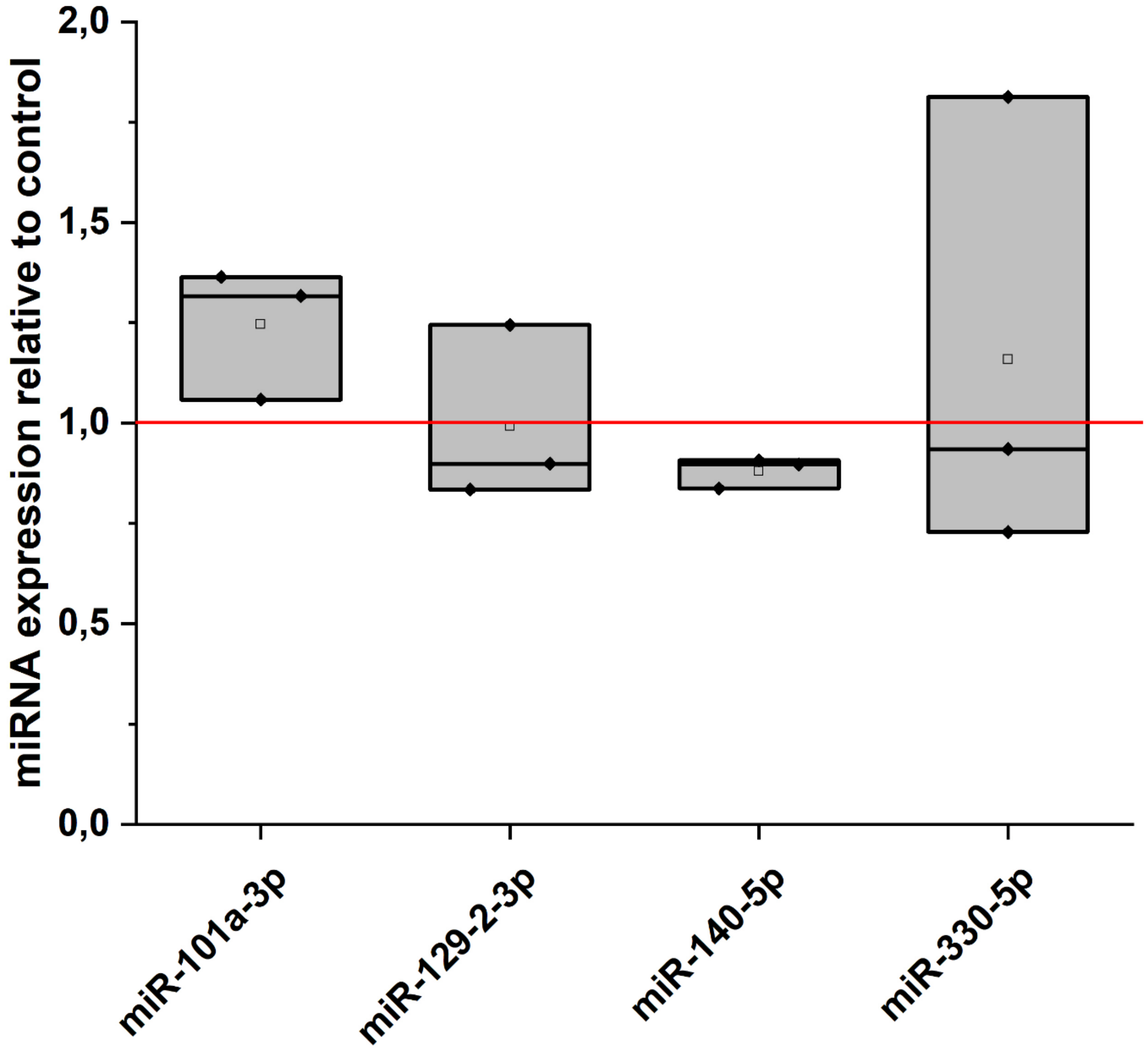

Supplement: Supplementary file 4 — (PDF 128 kb) [file 12192_2023_1371_MOESM4_ESM.pdf]
